# Predictors of survival in triple-negative breast cancer patients treated with neoadjuvant chemotherapy, surgery, and radiotherapy

**DOI:** 10.3389/fonc.2026.1880437

**Published:** 2026-08-04

**Authors:** Sha-li Shao, Wei Shi, Li Zhang, Qi-xian Zhang, Jin Meng, Xiao-mao Guo, Xiao-li Yu, Qiu Tang

**Affiliations:** 1 Department of Radiation Oncology, Women’s Hospital, School of Medicine, Zhejiang University, Hangzhou, Zhejiang, China; 2 Zhejiang Provincial Key Laboratory of Precision Diagnosis and Therapy for Major Gynecological Diseases, Women’s Hospital, Zhejiang University School of Medicine, Hangzhou, China; 3 Zhejiang Provincial Clinical Research Center for Obstetrics and Gynecology, Hangzhou, Zhejiang, China; 4 Department of Radiation Oncology, Fudan University Shanghai Cancer Center, Shanghai, China; 5 Department of Oncology, Shanghai Medical College, Fudan University, Shanghai, China; 6 Shanghai Clinical Research Center for Radiation Oncology, Shanghai, China; 7 Shanghai Key Laboratory of Radiation Oncology, Shanghai, China

**Keywords:** basal-like immune-suppressed, immunohistochemistry-based molecular subtyping, locoregional recurrence, lymph node ratio categories, Triple-negative breast cancer

## Abstract

**Background:**

Triple-negative breast cancer (TNBC) is known for its heterogeneity. Due to the small sample sizes in prior studies, there is limited understanding of whether different molecular subtypes have distinct predictive values following comprehensive treatment.

**Methods:**

Females with TNBC treated with neoadjuvant chemotherapy (NAC), surgery, and radiotherapy from 2019 to 2022 were retrospectively analyzed. Disease-free survival (DFS) and overall survival (OS) were evaluated using the Kaplan-Meier method to compare the basal-like immune-suppressed (BLIS) and non-BLIS subtypes. Univariate and multivariate Cox analyses were employed to identify prognostic factors for DFS.

**Results:**

The median follow-up time was 34 months. The 4-year DFS rates for non-BLIS and BLIS subtypes were 74.8% and 56.1%, respectively (P = .035). Independent predictors of DFS included molecular subtype (HR = 2.071, P = .034) and ypN status (HR for ypN1-2 = 4.891, P = .018; ypN3 = 7.444, P = .008). Additionally, both nodal stage and lymph node ratio categories (LNRC) were negatively correlated with DFS in both subgroups (P <.001 for both). Moreover, in the BLIS group, there was a statistically significant difference between low and high LNR (P < 0.05), while in the non-BLIS group, DFS differed significantly across subgroups with low, intermediate, or high LNRC (P < 0.05).

**Conclusions:**

Molecular subtyping plays a crucial role in prognosis, with the non-BLIS subtype showing more favorable outcomes. The presence of residual nodal disease post-NAC helps distinguish between favorable and unfavorable outcomes in different TNBC subtypes. These findings underscore the importance of developing more individualized radiotherapy strategies following NAC for each molecular subtype.

## 1 Introduction

Triple-negative breast cancer (TNBC), characterized by the absence of estrogen receptors (ER), progesterone receptors (PR), and human epidermal growth factor receptor 2 (HER2), accounts for 15-20% of newly diagnosed breast cancer cases (1). The local-regional recurrence (LRR) of the TNBC subtype showed a double-peak pattern with the first peak at 1–2 years after surgery (2). Early recurrence is a prominent feature of TNBC compared to other breast cancer subtypes.

The heterogeneity and aggressive nature of TNBC contribute to its poor prognosis. Numerous studies have investigated molecular subtyping in TNBC, classifying it into distinct subgroups (3, 4). Recently, Jiang et al. (5) classified TNBC into mRNA expression-based subtypes based on the multiomic analysis of 465 TNBC samples, introducing immunohistochemical biomarkers to streamline classification and enhance clinical applicability (6).

Neoadjuvant chemotherapy (NAC) is the standard treatment for locally advanced breast cancer (7) and is widely used in high-risk patients (HER2+ or triple-negative) (8). NAC serves to downstage tumors and assess their sensitivity to chemotherapy (9). Achieving a pathologic complete response (pCR) following NAC is associated with improved disease-free survival (DFS) and overall survival (OS) (10). However, it remains unclear if radiotherapy (RT) regimens should be adjusted based on primary tumor and lymph node status after NAC. Clinical trials of reducing the extent of axillary surgery (NCT01901094) and RT (11) in patients who achieved pCR after NAC are ongoing. While differences in radiosensitivity among breast cancer subtypes have been observed (12), current RT volume guidelines have not been modified. As personalized cancer care becomes increasingly important, treatment strategies that incorporate risk stratification and tumor characteristics are critical. Additionally, understanding the recurrence patterns of various molecular subtypes may aid in optimizing surveillance strategies.

In this study, all TNBC patients underwent neoadjuvant chemotherapy, surgery, and radiotherapy. We classified them into subtypes based on immunohistochemical markers. We hypothesized that different subtypes have different survival outcomes and aimed to elucidate the patterns of treatment failure and risk factors for prognosis.

## 2 Methods

### 2.1 Patients

One hundred and thirty-nine breast cancer patients treated with NAC followed by mastectomy and postmastectomy radiotherapy (PMRT) between November 2019 and March 2022 at Fudan University Shanghai Cancer Center were included. The study was approved by the institutional ethics committee. Clinicopathologic details (tumor location, clinical stage including tumor size and lymph node status date of surgery, grade, lymphovascular invasion status, pathological stage including residual tumor size and number of positive nodes) and treatment information (chemotherapy regimens, surgical approach, adjuvant therapy RT dose and fractionation) were extracted from patient’s medical records. The status of ER, PR, and HER2 was determined by immunohistochemical analysis. HER2 amplification was tested by fluorescence *in situ* hybridization (FISH). According to the ASCO/CAP guideline, the cutoff for ER/PR negativity in IHC was positive stained cells less than 1%. The stain score of HER2 under 1 or absence of HER2 gene amplification by FISH was considered HER2 negativity. IHC markers (AR, CD8, FOXC1, and DCLK1) were used to classify TNBCs into four IHC-based subtypes. Patients with an initial diagnosis of distant metastases, occult breast carcinoma or a history of contralateral breast cancer were excluded from this study.

### 2.2 IHC-based molecular subtyping

**Figure 1:**
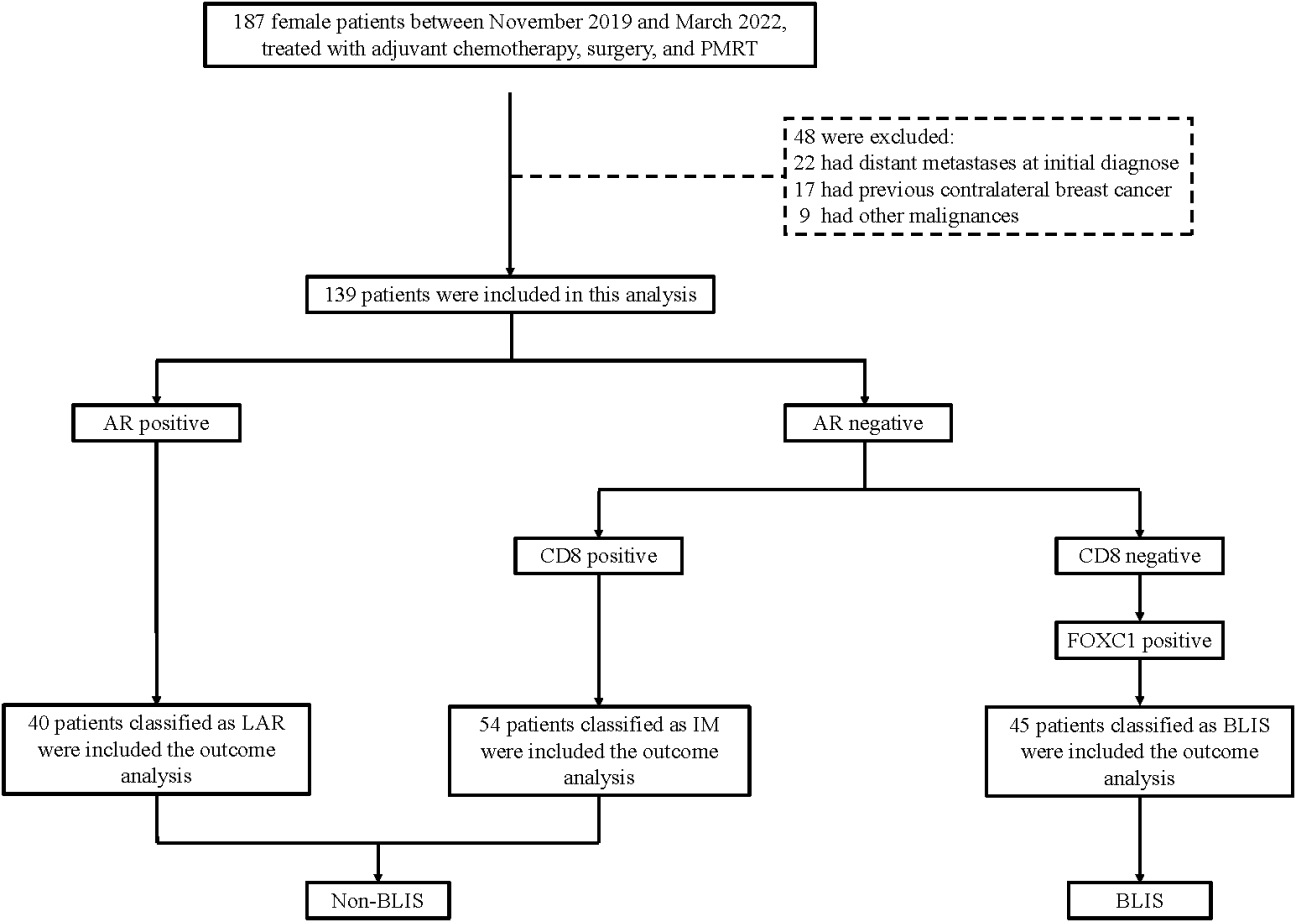
Flow diagram describing breast cancer cases from Fudan University Shanghai Cancer Center.

The IHC-based molecular subtyping classification was according to FUSCC criteria. The IHC cut-off for AR, CD8, FOXC1, and DCLK positive status was ≥10% positive tumor cells. TNBCs were classified into the four subtypes: (a) IHC-based luminal androgen receptor (IHC-LAR): AR-positive (+); (b) IHC-based immunomodulatory (IHC-IM): AR-negative (−) and CD8 (+); (c) IHC-based basal-like immune-suppressed (IHC-BLIS): AR (−), CD8 (−), and FOXC1 (+); (d) IHC-based mesenchymal (IHC-MES): AR (−), CD8 (−), FOXC1 (−), and DCLK1(+). The IHC-MES group was not included in this study (n = 0). The LAR and IM were combined into the non-BLIS group for further analysis (**Figure 1**).

### 2.3 Systemic therapy and surgery

In a taxane-based regimen, EC-P (epirubicin and cyclophosphamide followed by paclitaxel) and PC (paclitaxel and carboplatin) were the most common regimens in NAC. TAC (docetaxel, doxorubicin, and cyclophosphamide) and EC were also applied in preoperative systemic therapy.

Surgery depended on tumor response and patients’ willingness. All patients received mastectomy. Axillary lymph node dissection was routinely performed (n= 132, 95.0%), with a median of 18 dissected lymph nodes (range, 2-41). Lymph node ratio (LNR) was calculated by dividing the number of positive nodes by the total number of surgically removed nodes, with a median of 0.22 in the node-positive subgroup (range 0.04-1.0). The calculated values were divided into LNR categories (LNRC):LNR1, LNR = 0; LNR2, 0.01≤LNR ≤ 0.2; LNR3, 0.2<LNR ≤ 0.65; LNR4, LNR>0.65 (13).

The adjuvant chemotherapy mainly depended on the dose and regimens of NAC and the tumor response. GP (gemcitabine and cisplatin), PC, and EC-P were commonly applied in postoperative therapy.

### 2.4 Radiation therapy

For patients with indications of adjuvant chemotherapy, PMRT should be performed after completion of adjuvant chemotherapy, for those without, PMRT should be initiated within 8 weeks after surgery on the premise of recovery of upper limb function. Intensity-modulated radiation therapy (IMRT) planning was used. The delineation of the clinical target volume (CTV) is mostly based on the guidelines of Radiation Therapy Oncology Group (RTOG) and/or the European Society Therapeutic Radiation Oncology (ESTRO). The CTV included the chest wall and regional nodes. Regional nodal irradiation (RNI) was based on lymph nodal status and mainly includes supraclavicular, undissected axillary with or without the internal mammary nodes (IMN). Patients with medially located tumors and positive lymph nodes were more frequently treated with internal mammary node irradiation (IMNI). The undissected axilla included interpectoral nodes and axillary lymph nodes retained after axillary lymph node dissection. For patients with pathologically negative lymph nodes after NAC, it is up to the individual radiation oncologist to decide whether the RNI can be omitted. Conventional fractionation (50Gy/25Fx) and hypofractionated radiation therapy (42.56Gy/16Fx) were delivered in this study. An additional boost of 10–16 Gy was applied in patients with pathologically confirmed and/or highly suspected residual disease on image examination. The organs at risk included the spinal cord, heart, bilateral lungs, contralateral breast, humeral head, and thyroid.

### 2.5 Patient follow-up and outcomes

The frequency of required follow-up visits depends on the time from surgery: every three months for the first two years post-surgery, twice a year thereafter, and once a year after five years. Follow-up visits mainly included physical examinations, laboratory tests, and imaging examinations. Any suspicious lesions of metastasis were detected during follow-up, patients underwent PET-CT scanning for comprehensive assessment. Importantly, all suspected metastatic lesions were subjected to pathological biopsy, and the final diagnosis was exclusively based on histopathological findings. LRR was defined as breast cancer recurrence ipsilateral chest wall or regional lymph nodes, such as ipsilateral axillary, supraclavicular, or IMN. DFS was defined as the interval from the date of surgery to LRR, distant metastases (DM), or death. OS was defined as the time from surgery to death. No residual invasive disease in the breast and axilla was defined as pCR.

### 2.6 Statistical analysis

Basic clinicopathological information was presented as frequencies and percentages and analyzed using the Chi-square test or Fisher’s exact test. Kaplan-Meier curves were used to analyze the survival probability for DFS and OS and were compared using the log-rank test. The Cox regression model was used for the analysis of DFS. SPSS version 24.0 (SPSS, Chicago, IL, USA) and R version 4.1.3 were used for statistical analyses. Two-sided p values <.05 indicate statistical significance.

## 3 Results

### 3.1 Patient and treatment characteristics

This study retrospectively analyzed 139 breast cancer patients, with a median age of 47 years (range, 25-82). Detailed patient characteristics and treatment information are summarized in **Table 1**. Most patients (93.5%) underwent taxane-based preoperative therapy. IHC analysis classified 94 patients into the non-BLIS group and 45 into the BLIS group. In addition to the lower pCR rate and higher adjuvant chemotherapy rate in the BLIS group, other characteristics were balanced between the two groups.

**Table 1:** Baseline clinicopathologic and treatment characteristics of patients.

Characteristics		Non-BLIS (n=94) n (%)	BLIS (n=45) n (%)	Total (n=139) n (%)	*P* value
Age(y)	≤45	34 (36.2)	23 (51.1)	57 (41.0)	.094
	>45	60 (63.8)	22 (48.9)	82 (59.0)	
Location	Left	49 (52.1)	28 (62.2)	77 (55.4)	.263
	Right	45 (47.9)	17 (37.8)	62 (44.6)	
Clinical T stage	1-2	54 (57.4)	26 (57.8)	80 (57.6)	.971
	3-4	40 (42.6)	19 (42.2)	59 (42.4)	
Clinical N stage	0-1	39 (41.5)	14 (31.1)	53 (38.1)	.239
	2-3	55 (58.5)	31 (68.9)	86 (61.9)	
pCR	Yes	21(22.3)	3(6.7)	24(17.3)	.**022**
	No	73(77.7)	42(93.3)	115(82.7)	
ypT stage	0	25(26.6)	7(15.6)	32(23.0)	.083
	1-2	66(70.2)	33(73.3)	99(71.2)	
	3-4	3(3.2)	5(11.1)	8(5.8)	
ypN stage	0	40 (42.6)	18 (40.0)	58 (41.7)	.943
	1-2	41 (43.6)	21 (46.7)	62 (44.6)	
	3	13 (13.8)	6 (13.3)	19 (13.7)	
Lymph-vascular invasion	Yes	39 (41.5)	23 (51.1)	62 (44.6)	.559
	No	39 (41.5)	16 (35.6)	55 (39.6)	
	Unknown	16 (17.0)	6 (13.3)	22 (15.8)	
Grade	II	18(19.1)	9(20.0)	27(19.4)	.489
	III	31(33.0)	19(42.2)	50(36.0)	
	Unknown	45(47.9)	17(37.8)	62(44.6)	
NAC type	Anthracycline	6(6.4)	1(2.2)	7(5.0)	.361
	Taxane	35(37.2)	23(51.1)	58(41.7)	
	Anthracycline and Taxane	51(54.3)	21 (46.7)	72(51.8)	
	Other	2(2.1)	0	2(1.4)	
Adjuvant therapy	Yes	70(74.5)	41(91.1)	111(79.9)	.**022**
	No	24(25.5)	4(8.9)	28(20.1)	
Adjuvant therapy type	Anthracycline	7(10.0)	3(7.3)	10(9.0)	.630
	Taxane	12(17.1)	8(19.5)	20(18.0)	
	Anthracycline and Taxane	9(12.9)	2(4.9)	11(9.9)	
	Capecitabine*	28(40.0)	21(51.2)	49(44.1)	
	Other	14(20.0)	7(17.1)	21(18.9)	

BLIS, basal-like immune-suppressed; N, lymph node metastasis; NAC, neoadjuvant chemotherapy; pCR, pathologic complete response; T, tumor size.

* Capecitabine 1250 mg/m2 twice a day on day 1-14 every 3wk for 6-8 cycles or capecitabine 650 mg/m2 twice a day by month for 1 year

Bold values P < 0.05.

### 3.2 Patterns of recurrence

The patterns of tumor recurrence are presented in **Table 2**. In total, recurrence was identified in 41 patients through imaging and pathological examination. Among the 19/41 patients with LRR, 11 had isolated LRR, while 8 experienced LRR with concurrent distant metastases, and no isolated out-of-field relapse was observed. Distant metastasis was the main event of tumor recurrence. Distant metastases occurred in 30 patients across the cohort. In both non-BLIS and BLIS subtypes, the most common sites for metastasis are the lung and bone. The number of patients presenting with concurrent multisite metastases was 7 in the non-BLIS group and 9 in the BLIS group, respectively. The DM rates were 33.3% in the BLIS group and 16% in the non-BLIS group.

**Table 2:** Patterns of recurrence.

Characteristics	Non-BLIS (n=94) n (%)	BLIS (n=45) n (%)
Any breast cancer recurrence	23 (24.5)	18 (40.0)
Local-regional recurrence (ipsilateral)	11(11.7)	8 (17.8)
Isolated	8 (8.5)	3 (6.7)
With simultaneous distant metastases	3 (3.2)	5 (11.1)
Local recurrence (ipsilateral)	7 (7.4)	3 (6.7)
Regional recurrence (ipsilateral)	7 (7.4)	3 (6.7)
Axillary	2 (2.1)	1 (2.2)
Supraclavicular	4 (4.3)	2 (4.4)
Internal mammary	1 (1.1)	0
Distant metastases	15 (16.0)	15 (33.3)

BLIS, basal-like immune-suppressed

### 3.3 Survival

**Figure 2:**
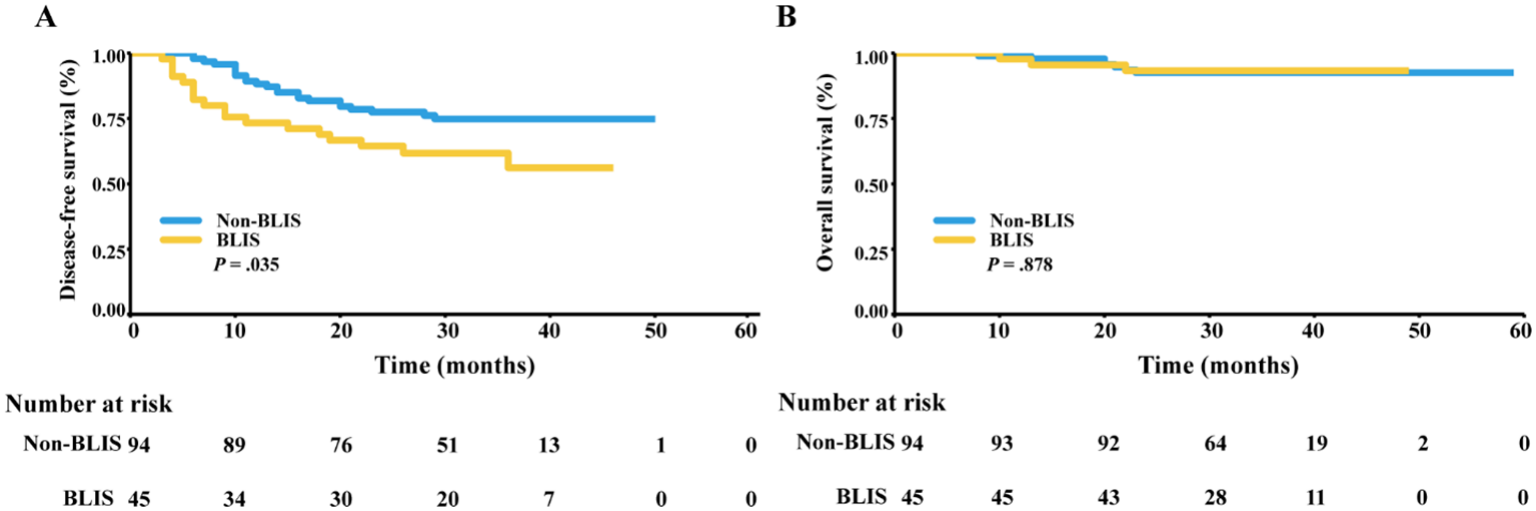
Survival by Kaplan–Meier analysis among patients by different molecular subtypes. **(A)** Disease-free survival (DFS). **(B)** Overall survival (OS).

**Figure 3:**
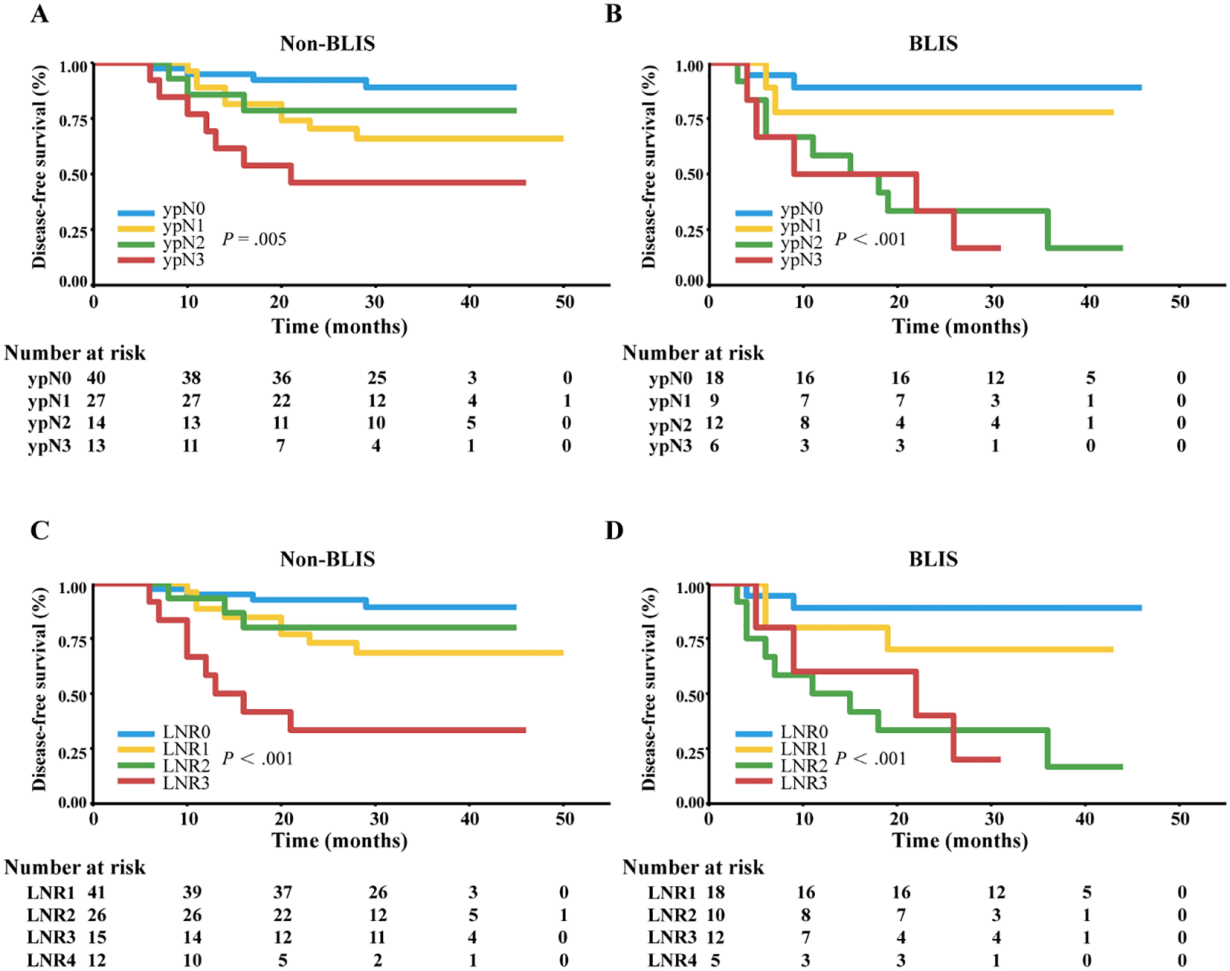
Comparison of disease-free survival by Kaplan–Meier analysis by different ypN nodal stage and lymph node ratio (LNR) in different molecular subtypes. **(A)** ypN staging in non-BLIS subtype. **(B)** ypN staging in BLIS subtype. **(C)** lymph node ratio categories (LNRC) in non-BLIS subtype. **(D)** LNRC in BLIS subtype. (LNR1, LNR = 0; LNR2, 0.01≤LNR ≤ 0.2; LNR3, 0.2<LNR ≤ 0.65; LNR4, LNR>0.65).

The median follow-up period was 34 months. In total, 10 patients died. The 4-year DFS and OS rates were 68.5% and 92.8%. The 4-year DFS rate for the BLIS subtype was 56.1%, compared to 74.8% in the non-BLIS subtype (*P* = .035). The improved DFS in the non-BLIS subtype did not result in a longer OS, with 4-year OS rates of 92.6% and 93.3% in the non-BLIS and BLIS subtypes, respectively (*P* = .878) (**Figure 2**). Univariate and multivariate analysis results for DFS in the entire cohort are summarized in **Table 3**. Multivariate analysis identified IHC-based molecular subtyping as an independent prognostic factor for DFS (hazard ratio [HR] = 2.071; 95% CI, 1.068-3.990; *P* = .034). ypT and ypN staging were also independent prognostic factors for DFS (*P* <.05). Significant differences were observed between ypN1 and both ypN2 and ypN3 within the BLIS group (*P* <.05). In the non-BLIS subgroup, only the comparison between ypN0 and ypN3 showed statistical significance (*P* <.05). LNRC was similarly inversely correlated with DFS. The difference between LNR1 and LNR4 in the BLIS group was statistically significant (*P* <.05). In the non-BLIS group, significant differences were observed among all subgroups (*P* <.05) (**Figure 3**).

**Table 3:** Univariate and multivariate Cox regression analysis of DFS.

Characteristics		UVA (DFS)		MVA (DFS)	
		HR 95% CI	*P* value	HR 95% CI	*P* value
Subtype	Non-BLIS	1		1	
	BLIS	1.914 (1.032-3.547)	**.039**	2.071 (1.058-4.054)	**.034**
Age	≤45y	1		1	
	>45y	1.082 (0.578-2.027)	.805	1.292 (0.592-2.822)	.520
Tumor location	Left	1		1	
	Right	0.888 (0.477-1.654)	.709	0.846 (0.422-1.694)	.637
Clinical T stage	1-2	1		1	
	3-4	1.548 (0.838-2.858)	.163	1.616 (0.766-3.407)	.207
Clinical N stage	0-1	1		1	
	2-3	1.866 (0.934-3.728)	.077	1.146 (0.539-2.436)	.723
ypT	0	1		1	
	1-2	1.654 (0.686-3.989)	.263	1.047 (0.296-3.706)	.943
	3-4	7.704 (2.469-24.041)	**< .001**	4.820 (0.962-24.156)	.056
ypN	0	1		1	
	1-2	4.147 (1.687-10.191)	**.002**	4.891 (1.306-18.309)	**.018**
	3	8.553 (3.202-22.846)	**< .001**	7.444 (1.676-33.063)	**.008**
Lymph-vascular invasion	Positive	1		1	
	Negative	0.252 (0.110-0.578)	**.001**	0.698 (0.245-1.990)	.502
	Unknown	0.695 (0.302-1.596)	.695	1.930 (0.718-5.189)	.193
Grade	II	1		1	
	III	1.002 (0.447-2.250)	.996	1.519 (0.563-4.097)	.409
	Unknown	0.696 (0.304-1.590)	.390	1.430 (0.555-3.686)	.459
pCR	No	1		1	
	Yes	0.219 (0.053-0.908)	**.036**	1.052 (0.123-8.968)	.963
Adjuvant	No	1		1	
	Yes	1.174 (0.636-2.168)	.608	1.087 (0.552-2.141)	.808

BLIS, basal-like immune-suppressed; CI, confidence interval; DFS, disease-free survival; HR, hazard ratio; MUV, multivariate analysis; N, lymph node metastasis; pCR, pathologic complete response; T, tumor size; UVA, univariate analyse

Bold values P < 0.05.

## 4 Discussion

This study evaluated the impact of IHC-based molecular subtyping on locally advanced TNBC patients treated with NAC. The analysis identified IHC-based molecular subtyping and ypN stage as independent prognostic factors, with the BLIS subtype being linked to poorer survival outcomes. The assessment of residual cancer burden in axillary nodes showed that both ypN stage and LNRC were associated with survival outcomes.

Biological subtypes have been considered as risk factors for clinical survival. Previous studies showed that TNBC is linked to a poorer prognosis, with higher rates of LRR compared to HER-2+ or HR+ subtypes (14, 15). These findings highlight the complex relationship between biological subtypes and clinical outcomes. TNBC is a heterogeneous disease with varied progression and prognosis. Molecular subtyping based on genomic and transcriptomic profiles offers a theoretical foundation for personalized TNBC treatment (5). IHC-based molecular subtyping aligns with genetic expression, offering comparable prognostic value (16). In this study, molecular subtyping emerged as an independent prognostic factor for DFS in patients treated with NAC, mastectomy, and PMRT, with the BLIS subtype showing worse outcomes. However, compared to non-BLIS subtypes, the BLIS subtype did not show a statistically significant difference in OS. This discrepancy may be attributed to the relatively short follow-up period of the current study. Although TNBC is characterized by high malignancy, favorable survival outcomes can still be achieved with standardized treatment. Consequently, continued follow-up to draw more definitive and scientifically robust conclusions. Our findings further support that molecular subtyping is linked to DFS in breast cancer and provide a deeper understanding of TNBC’s heterogeneity.

The increasing use of NAC presents challenges in determining the best approach to local and regional management. Since treatment decisions rely heavily on clinical and pathological factors, the varying responses to NAC complicate choices regarding optimal assessment techniques, surgical timing, and the application of PMRT (17). pCR is recognized as a prognostic indicator. Patients who achieve pCR tend to have better survival rates compared to those with remaining tumors (18, 19). TNBC patients are more likely to achieve pCR following NAC compared to luminal subtypes (20). However, while pCR was significant in univariate analysis, it did not independently predict distant metastasis in this study. Our study showed a pCR rate of 17.3%, lower than the reported 30%-40% in the literature (19). This discrepancy may be due to our stricter definition of pCR, which required the absence of residual tumors in both the breast and axilla. Identifying additional prognostic risk factors for patients who do not achieve pCR is critical. Both univariate and multivariable analyses indicated that pathological N stage is linked to distant metastasis, aligning with findings from other studies (21, 22). However, it remains debated whether lymph node status changes following NAC should influence adjustments to radiotherapy regimens. Our findings revealed that 42% of TNBC patients became lymph node-negative after NAC. Previous retrospective studies suggest that PMRT improves local control and survival in ypN0 breast cancer patients (23), while other research shows that omitting PMRT in ypN0 patients post-NAC does not increase the risk of LRR (24). A meta-analysis found that PMRT reduced LRR but had no significant impact on DFS or OS in patients with Stage III ypN0 breast cancer (25). Our earlier research indicated that the BLIS subtype has a higher risk of LRR (unpublished). Therefore, decisions to omit PMRT in these patients should be made carefully, considering the tumor’s biology.

While extensive lymph node dissection has shown survival benefits in breast cancer patients, the optimal number and extent of dissections remain a subject of debate. LNR has been identified as a valuable prognostic marker for breast cancer patients following NAC (26). In our study, we assessed the prognostic significance of LNR in patients with locally advanced TNBC across various molecular subtypes. The results showed that increasing LNR was associated with worse DFS regardless of the non-BLIS subtype or BLIS subtype, which is consistent with the previous finding (27). An ongoing clinical trial (NSABP B-51/RTOG 1304) is currently addressing the role of regional lymph node radiotherapy.

LVI is commonly observed in patients with TNBC. Studies have shown that patients with LVI have a higher risk of developing distant metastases and generally face poorer survival outcomes (28, 29). While LVI did not independently predict outcomes in our study, it significantly increases the risk of distant metastasis in patients with aggressive tumor biology. More attention and individual treatment are needed in those cases.

Shiv Dasai et al. (30) found no significant differences in locoregional outcomes among patients who received postmastectomy radiotherapy (PMRT) at various time points. Another research, however, discovered that PMRT initiation within 8 weeks from surgery had a beneficial impact on clinical outcomes, with patients had better DFS and OS (31). In the present study, the median time to recurrence was 41.1 months for non-BLIS subtypes and 31.8 months for BLIS subtypes. Considering the more aggressive nature of the BLIS subtype, the timing of PMRT initiation after surgery is particularly important. PMRT should not be delayed for those patients who are “moderately high risk” - women diagnosed with BLIS subtype and advanced T and/or N stage.

This is the first study to evaluate the prognostic significance of immunohistochemistry (IHC)-based molecular subtyping in TNBC patients treated with NAC, surgery, and PMRT. However, the sample size of this study is limited, especially the lack of MES subtype. A treatment-stratified survival analysis was not feasible owing to small and unbalanced subgroups (e.g., only 4 BLIS patients did not receive adjuvant therapy) and confounding by indication, as adjuvant therapy was largely determined by the response to NAC; this question warrants investigation in a larger multi-center cohort. Being a retrospective study, selection bias is a potential limitation. The patients in this study were all from a single institution, and future research should include participants from multiple institutions to validate these findings. Moreover, longer follow-up periods would yield more comprehensive and meaningful data on this issue.

## 5 Conclusion

In conclusion, clinical outcome differentiation based on molecular subtypes offers new insights into the treatment of TNBC. Identifying high-risk factors enables the distinction of patient subgroups with unfavorable DFS. For these high-risk patients, tailored and intensified treatment strategies are essential.

## Data Availability

The raw data supporting the conclusions of this article will be made available by the authors, without undue reservation.

## Glossary

AR: Androgen receptor
BLIS: Basal-like immune-suppressed
CF: Conventional fractionation
CTV: Clinical target volume
DCLK1: Doublecortin-like kinase 1
DFS: Disease-free survival
DM: Distant metastases
EBCTCG: Early Breast Cancer Trialists’
ER: Estrogen receptor
FISH: Fluorescent in Situ Hybridization
FOXC1: Forkhead box transcription factor C1
HER2: Human epidermal growth factor receptor 2
HF: Hypofractionated fractionation
IHC: Immunohistochemical
IHC-BLIS: IHC-based basal-like immune-suppressed
IHC-IM: IHC-based immunomodulatory
IHC-LAR: IHC-based luminal androgen receptor
IHC-MES: IHC-based mesenchymal-like
IM: Immunomodulatory
IMN: Internal mammary node
IMNI: Internal mammary node irradiation
IMRT: Intensity-modulated radiation therapy
LAR: Luminal androgen receptor
LNR: Lymph node ratio
LNRC: Lymph node ratio categories
LR: Local recurrence
LRR: Locoregional recurrence
LVI: Lymphovascular invasion
MES: Mesenchymal-like
NAC: Neoadjuvant chemotherapy
OS: Overall survival
pCR: Pathological complete response
PMRT: Postmastectomy radiotherapy
PR: Progesterone receptor
PTV: Planning target volume
RNI: Regional nodal irradiation
RR: Regional recurrence
TNBC: Triple-negative breast cancer
VEGF: Vascular endothelial growth factor
